# COVID-19 in India: Epidemiological reflections from initial 170 million consecutive test results

**DOI:** 10.3389/fepid.2022.933820

**Published:** 2022-10-18

**Authors:** Rohan Lohia, Prabudh Goel, Jasmine Kaur, Sujeet Kumar, Minu Bajpai, Harpreet Singh

**Affiliations:** ^1^Clinton Health Access Initiative, New Delhi, India; ^2^Department of Pediatric Surgery, All India Institute of Medical Sciences, New Delhi, India; ^3^Department of Biomedical Informatics, Indian Council of Medical Research (ICMR), New Delhi, India; ^4^Centre for Proteomics and Drug Discovery, Amity Institute of Biotechnology, Amity University, Mumbai, India

**Keywords:** coronavirus – COVID-19, pandemic (COVID-19), epidemiological reflections, COVID positivity, gender, age

## Abstract

**Background:**

The Indian Council of Medical Research (ICMR) played a crucial role in streamlining testing and diagnosis, formulating guidelines, and devising management strategies during the COVID-19 pandemic. Additionally, ICMR designed and developed a comprehensive data management tool for collecting testing data in a standardized format from all laboratories across the country. The current report is a retrospective analysis of the testing data generated by the ICMR. The study's main objectives are to understand the probability of a person testing negative based on their age after an initial positive test and to assess the varied impact and duration of the disease in people of different age groups and genders.

**Methods:**

Anonymized data on the testing for COVID were analyzed. The P-to-P is the longest time interval between two consecutive positive tests for a patient without any negative test in between the positives. P-to-P_last_ is the time between the first positive and last positive test, as opposed to P-to-P, here we are looking at the first and last positive tests that might or might not be consecutive. P-to-N intervals is the time between the first positive and first negative test of a patient.

**Results:**

India conducted 170,914,170 tests during the study-period (until December 29, 2020). After excluding invalid test results and duplicates, there were 11,101,603 (6.5%) positive and 156,542,352 (93.5%) negative test-results performed upon 150,086,257 unique individuals. A negative-report following a positive-test was available in 12.69%. Nearly three-fourths of the cases (78.29%) belonged to the working-age group (18–60 years). The proportion of patients >50 years old has risen from 26.06 to 35.03%, with a steep rise beyond September 2020. Gender-ratio among the positives was 1.73:1 which was neutral in neonates < 7-days (age). The gender ratio was skewed in-favor-of males in the initial months with a reverse trend thereafter and with increasing age of patients. The mean P-to-P, P-to-P_last_, and P-to-N durations were 12.7 + 4.3, 13.3 + 4.6, and 14.2 + 4.9 days for individuals with P-to-P duration of 1–4 weeks. The probability of testing negative was 82 & 85% at 14 & 21 days after the first-positive-test respectively with no gender bias.

**Conclusions:**

The current study has highlighted some vital aspects of COVID-19 epidemiology in India. This study will add to the current understanding of the virus in the absence of pre- existing information on the novel virus and the disease per se.

## Introduction

The first case of COVID-19 (**CO**rona **VI**rus **D**isease; SARS-CoV-2) in India was reported on January 30, 2020 (1) in Thrissur, Kerala. As of December 29, 2020, India has reported 11,101,603 positives and 148,475 deaths, highest (cases) next only to the United States of America (2). The corresponding figures at world-level were 82,374,360 positives and 1,797,684 deaths (2). Being a novel virus, no published literature or milestones for guidance were available at inception. The year 2020 witnessed a steep upsurge in COVID-19 related publications which neared 90,000 on PUBMED (3) alone by the end of the year. However, enhancement of our understanding of the disease and related aspects of the disease is still awaited (4–7) .

ICMR played a key role in management of the coronavirus pandemic (8). Ever since the first case was detected in India, testing was initiated in a group of Virus Testing Laboratories (VRDLNs) across the country. The data was collected in excel sheets. However, as the number of cases increased, more testing methods like TrueNAT, CBNAAT, Rapid Antigen Testing were introduced. Also, a data management tool was developed and released for capturing the pandemic data (9, 10). The current report is a retrospective analysis of the testing data collected by the ICMR. The main objectives of the study are to understand the probability of a person to test negative based on their age after an initial positive test and to access the varied impact and duration of the disease in people of different age groups and genders.

## Materials and methods

### Data

It has been mandatory upon all testing facilities to report covid-test information to the ICMR portal in a pre-designed Specimen Referral Form (SRF) (11). Testing data was imported from the COVID-19 data-repository of the ICMR (9, 12, 13) and anonymized prior to use for the analysis. India's population and demographics data were used for comparison with the demographics of the tested population. The population data was imported from the UN statistics division website (14).

### Data analysis

Data was analyzed with the use of 'R' Programming language and R studio (15) (*version 1.3.1093 copyright 2009-2020 RStudio, PBC*) and Microsoft Excel (16) for Microsoft 365 MSO (*16.0.13530.20368*) 64-bit. Data corresponding to type of test, unique patient information ID, date of entry of test result, date of sample collection, test result, gender, and age were analyzed. For all, sub-analysis data was cleaned and filtered in R using the basic techniques: count, sum, MOD, concatenation of columns, addition, and subtraction. Following this, the data was exported to Excel. Given the size and complexity of the data set and the various factors considered, the data was analyzed individually for each parameter, such as gender and age, over time and the impact of different factors on the outcome of tests was analyzed.

To understand the **disease positivity**, a sub-group analysis was performed to identify the testing sequence(s) in patients who have been tested more than once with at least one positive report. Considering a positive test would be represented by 'P' and a negative test by 'N', those with P-P or P-N sequence in their test results were included in this analysis. Those with an N-P sequence in the absence of P-P or P-N were excluded. The following sequences were analyzed:

a. **P-to-P interval**: the duration between two consecutive positive tests without any intervening negative. In the event that a patient tested positive more than twice, the longest duration between two consecutive positive tests was included.
b. **P-to-P**_**last**_ **interval**: duration between first and last positive test before a negative test (if available).
c. **P-to-N interval**: the time duration between the first positive and first negative test of a patient.

The **Probability of Testing Negative 'n' Days After Testing Positive [P(n)]** is reflected by the proportion of people testing negative after 'n' days after the first positive test. This is only calculated for cases wherein a conclusive test is available 'n' days after the first positive.


$$\begin{array}{l} \text{P}\left(\text{n}\right)\text{ is calculated as}:\text{P}\left(\text{n}\right)\\ =\frac{\sum \text{Negative Test}{\text{s }}^{\text{′}}{\text{n}}^{\text{′}}\text{Days After First Positive}}{\sum \text{All Test}{\text{s}}^{{\;}^{\text{′}}}{\text{n}}^{\text{′}}\text{Days After First Positive}} \end{array}$$

## Results

During the study-period [January 27, 2020 - December 29, 2020], India conducted 170,914,170 (Table 1: A0) tests. Of these, 167,643,955 (Table 1: A1) tests have been considered valid and 3,270,215 (Table 1: A2) excluded due to leak or spillage, sample rejection by machine, inconclusive results, incomplete reporting, incongruent duplicates, or invalid entries (typographical errors). Among the valid tests, there were 11,101,603 (6.5%) (Table 1: B3) positive and 156,542,352 (93.5%) (Table 1: B4) negative test-results. Sub-group analysis of this group, however revealed the presence of 6,789,011 (Table 1: B2) duplicate test-results (same report upon same individual performed on the same day); the non-duplicate results were 160,854,944 (Table 1: B1). The duplicate results have been removed from the analysis as it is either a confirmatory test being done on the same individual or a duplicate entry of the test result, in either of the cases it does not add any meaningful conclusion to the main objectives of this study.

**Table 1:** A bird's eye view of the COVID-19 numbers from India (until December 29, 2020).

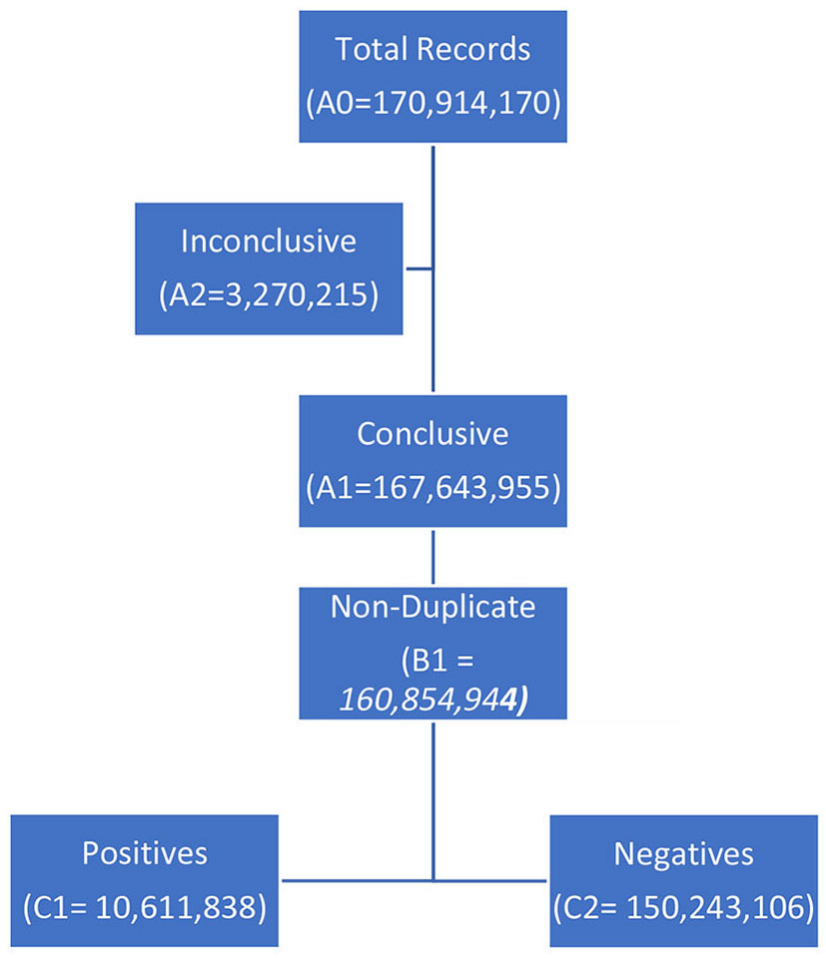

These tests (Table 1: B1) have been performed upon 150,086,257 (Table 1: C3) unique individuals. Each tested individual has undergone a mean of 1.072 ± 0.64 tests. It was observed that 8,741,043 (5.8%) (Table 1: C4) individuals were tested more than once (on different days) with an average of 2.232 ± 1.23 tests per individual. Furthermore, 0.91 and 0.042% of the individuals were tested more than 2- and 5-times, respectively.

Of the 160,854,944 (Table 1: B1) tests, positive reports were observed in 10,611,838 (Table 1: C1) (6.60%); however, within the 150,086,257 (Table 1: C3) unique individuals, positive reports were observed in 10,070,896 (Table 1: D1) (6.71%) while 140,015,361 (93.29%) never tested positive. Among the 8,741,043 (Table 1: C4) individuals with multiple tests, the incidence of positivity [positive: 2,174,115 (Table 1: D2) (24.87%) individuals; negative: 6,566,928 (75.13%)] was nearly **4-times (371%)** vis-à-vis those who were tested only once.

***A negative report following a positive test was available in only 12.69%*** (*n* = 1,277,538) (Table 1: D3) individuals (of 10,070,896 unique individuals who tested positive). There were 585,084 (Table 1: D4) (5.8%) individuals who tested positive more than once but were never concluded negative.

### Age-groups-positives

**Figure 1:**
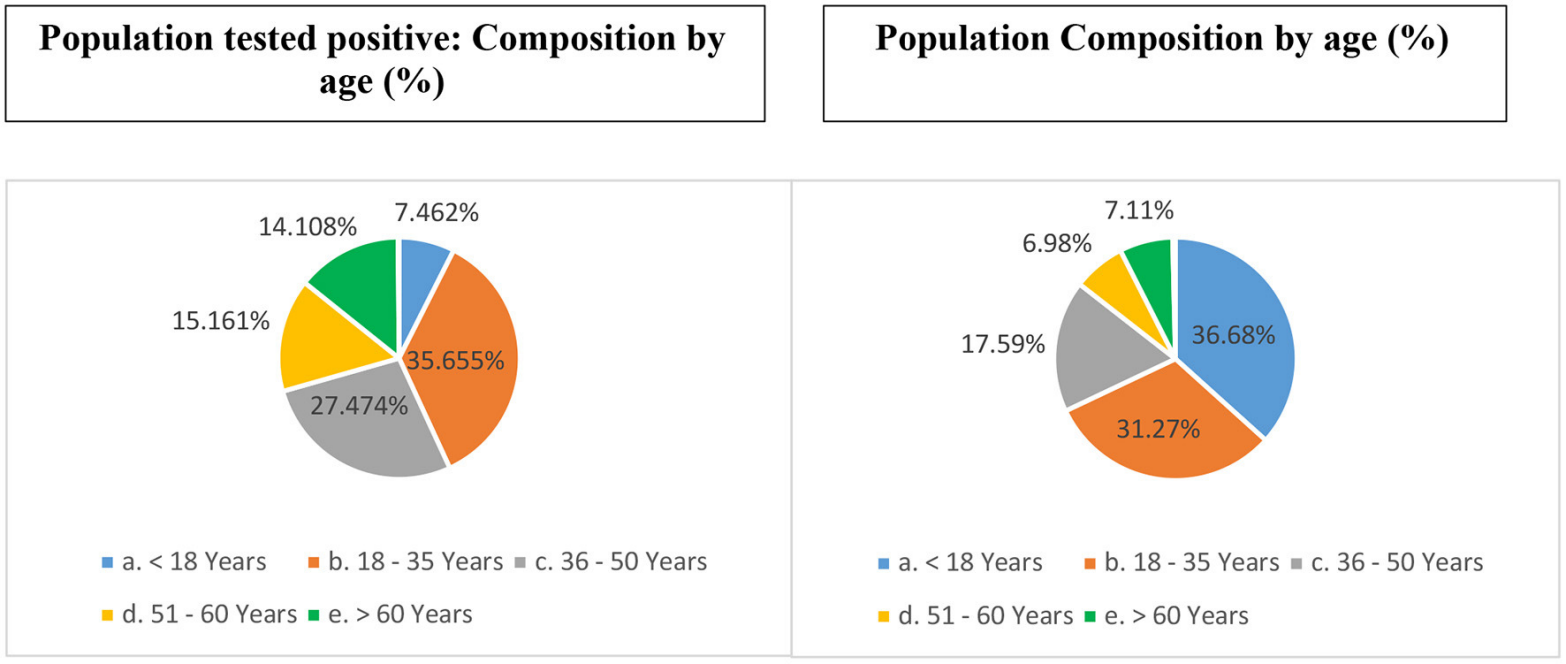
Age-based distribution of unique individuals who tested positive for COVID-19 from India (till December 29, 2020) (vis-à-vis proportional composition in the total population).

Individuals belonging to the age-group 18–35 contributed (31.4% of the total population) to more than one-third (~36%) of the caseload (Figure 1). Individuals < 18 years of age (36.7% of total population) accounted for 7.46% of the caseload. Individuals >60 years of age (7.1% of total population) accounted for 14.11% of the caseload.

Nearly three-fourth of the cases (78.29%) belonged to the working age-group (18–60 years) [c/f census proportion: 55.84%]. Children 10 years of age or younger accounted for 5.3% of the tests and 3.3% (*n* = 351,861) of the caseload [c/f census 22.32%]. Neonates and infants (c/f census 3.47%) accounted for 0.13 and 0.15% (*n* = 16,197) of the tests and the caseload, respectively.

**Figure 2:**
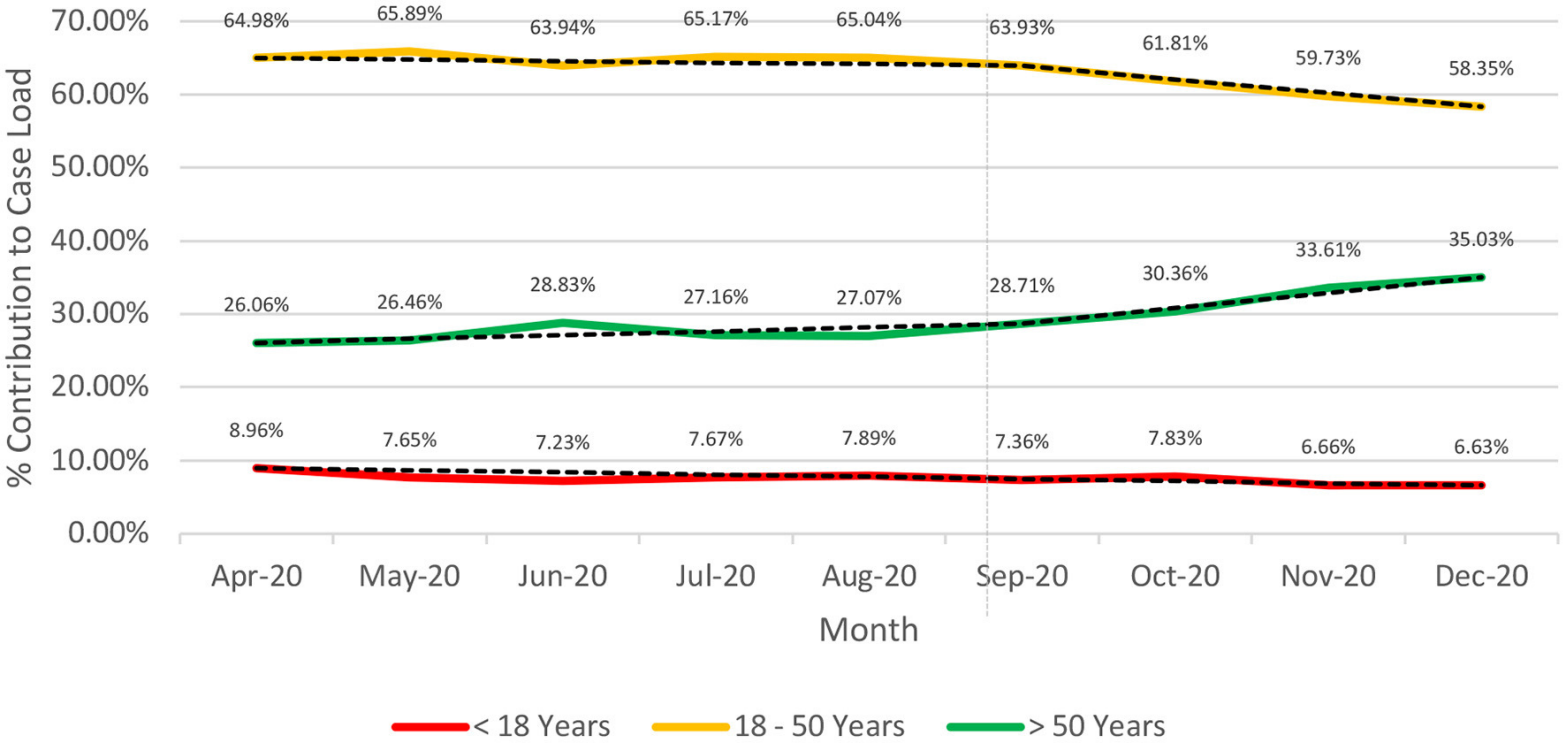
Longitudinal (April-Dec 2020). Age-based stratification of the caseload for COVID-19 from India (till December 29, 2020).

The proportion of cases >50 years of age has risen gradually over time (26.06 to 35.03%) since the inception of the pandemic with a steep rise beyond September 2020 (Figure 2). Contrarily, the proportion of cases in the age-group 18–50 years remained nearly constant until September; thereafter it has shown a decline from 63.93% (September 2020) to 58.35% (December 2020).

**Figure 3:**
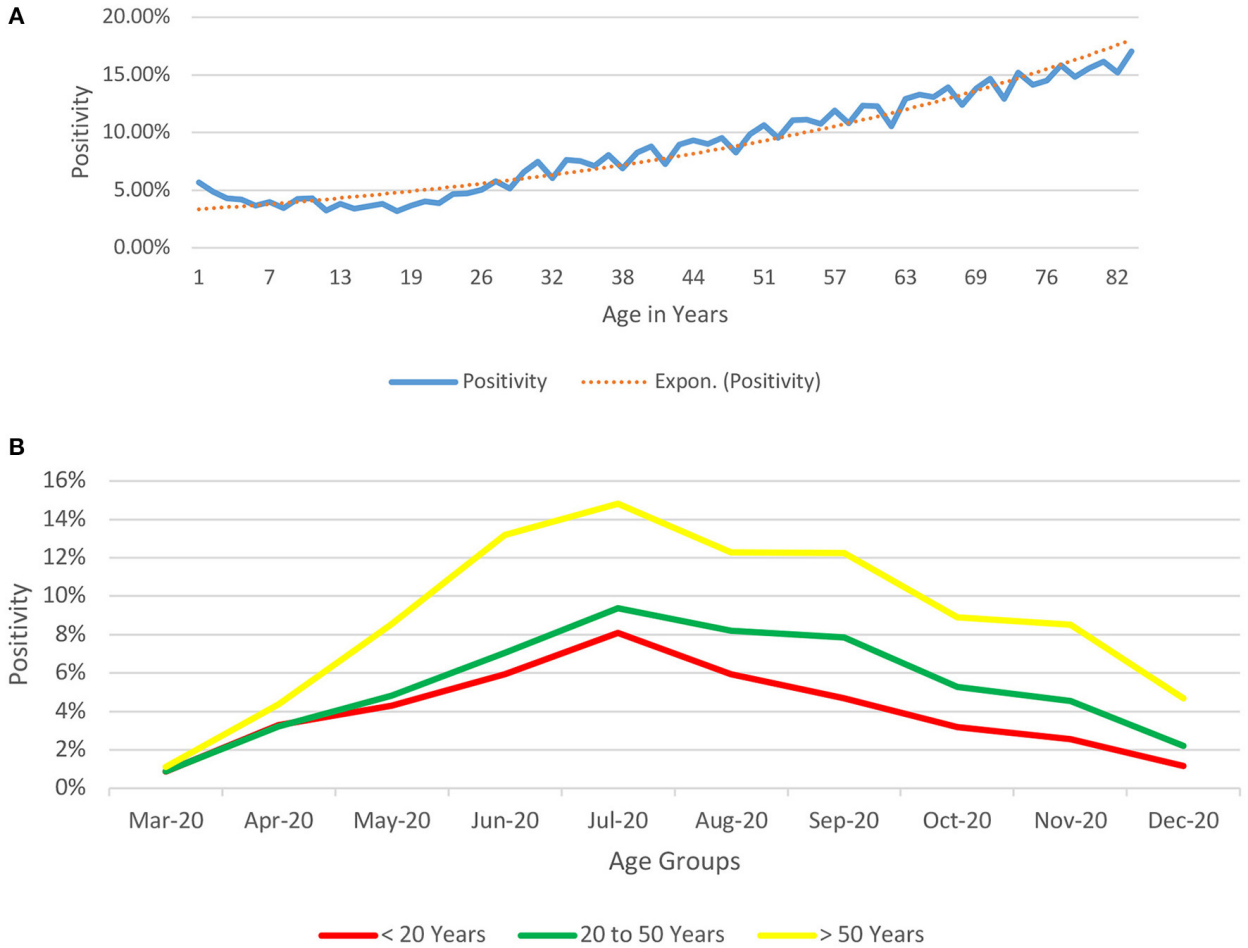
**(A)** Age-based Depiction of Positivity rates for COVID-19 from India (till December 29, 2020) (proportion of positive tests in a particular age-group to the total number of tests conducted in that age-group). The positivity rates (proportion of positive tests in a particular age-group to the total number of tests conducted in that age-group) depicts a bimodal pattern, being relatively high at the two extremes of age (5.67% for infants and neonates, 12.29% at 61 years of age and 16.16% at 81 years of age). Beyond infancy, the positivity declines till the age of 18 years (minimum positivity @ 3.18%) and shows a rising trend thereafter. **(B)** Age-based Depiction of Positivity rates over time for COVID-19 from India (till December 29, 2020) (proportion of positive tests in a particular age-group to the total number of tests conducted in that age-group over time). The positivity rates (proportion of positive tests in a particular age-group to the total number of tests conducted in that age-group) depicts that just like the observation in positivity rates increase with age and follow the same pattern throughout the period of the study until December 2020.

The positivity rates (proportion of positive tests in a particular age-group to the total number of tests conducted in that age-group) (Figure 3A) depicts a bimodal pattern.

### Gender-testing and positives

The male-to-female ratio among the total unique individuals tested was 1.5:1 while the sex ratio among those who tested positive was 1.73:1. Approximately, 6.66% (*n* = 6,724,130) (Table 1: D5) and 5.86% (*n* = 3,884,918) (Table 1: D6) of the males and females respectively who underwent a test, reported positive. Among those who underwent multiple tests, the male-to-female ratio was 1.94:1 (65.98%:34.01%).

The male-to-female ***case ratio*** was 1.02:1 (~ 1:1) in neonates 7 days of life or younger, 1.27:1 in neonates and infants and 1.18:1 for children less than 10 years of age compared to 1.73:1 for the entire population. The positivity rate was 3.99% in individuals who refrained from reporting their gender (0.042% of unique individuals).

**Figure 4:**
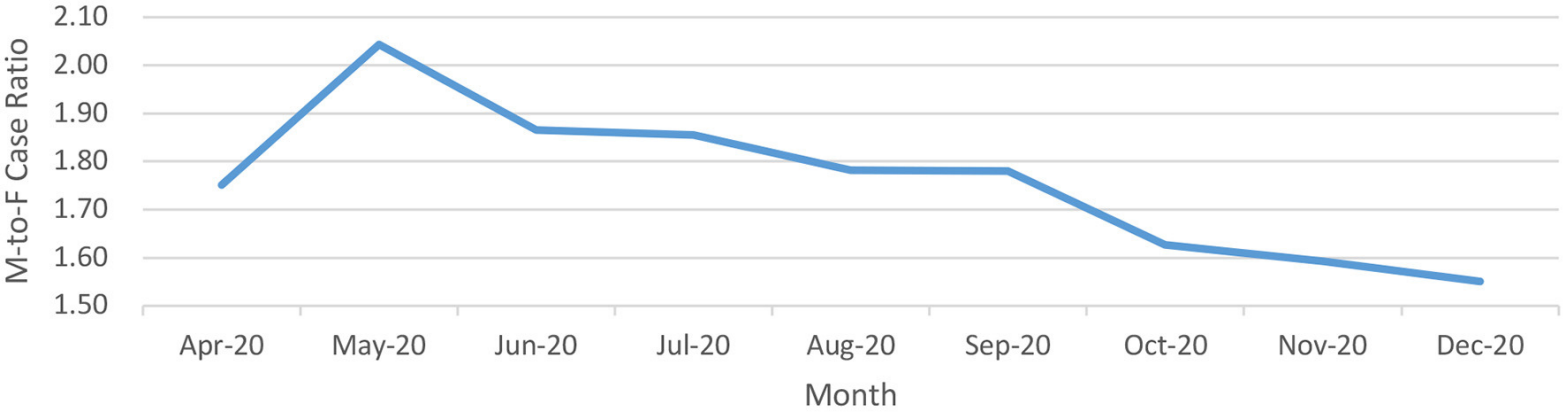
Male-to-Female ratio of positive cases over a longitudinal timeline for COVID-19 from India (until December 29, 2020).

The male-to-female ratio among the cases was skewed in favor of males (Figure 4) during the first few months of the pandemic (**1.98:1** during April-May 2020) with a trend toward rationalization from June 2020 onwards (**1.86:1** during June-July 2020, **1.78:1** during August- September 2020 and **1.60:1** during October-December 2020). The nadir of male-to-female during the study period has been observed during the month of December 2020.

### Age-gender sub-groups

The male-to-female ratio among those who tested positive for COVID-19 was skewed in favor of males with increasing age (Table 2). The male-to-female ratio was 1.25:1 in individuals less than 18 years of age compared to 1.78:1 in those who were 18 years or older.

**Table 2:** Age- and sex-based stratification of the individuals who tested positive for COVID-19 from India (until December 29, 2020).

**Gender**	**a. < 18 Years**	**b. 18–35 Years**	**c. 36–50 Years**	**d. 51–60 Years**	**e. > 60 Years**	**NA**	**Grand total**
Female	352,110 (44.46%)	1,362,284 (36%)	1,018,105 (34.92%)	592,809 (36.85%)	555,313 (37.09%)	4,297 (28.96%)	3,884,918 (36.61%)
Male	439,591 (55.51%)	2,420,336 (63.97%)	1,896,637 (65.05%)	1,015,617 (63.13%)	941,485 (62.88%)	10,464 (70.52%)	6,724,130 (63.36%)
NA	108 (0.01%)	572 (0.02%)	412 (0.01%)	231 (0.01%)	200 (0.01%)	62 (0.42%)	1,585 (0.01%)
Others	80 (0.01%)	448 (0.01%)	303 (0.01%)	186 (0.01%)	172 (0.01%)	16 (0.11%)	1,205 (0.01%)
**Total**	**791,889**	**3,783,640**	**2,915,457**	**1,608,843**	**1,497,170**	**14,839**	**10,611,838**

The observed male-to-female in children less than 10 years of age was 1.18:1 [c/f census 1.09:1], the same ratio was 1.37:1 in individuals 10–19 years of age [c/f census 1.11:1], 1.66:1 in individuals in 20–29 years of age [c/f census 1.05:1], and 2.01:1 in individuals in 30–39 years of age [c/f census 1.02:1]. Beyond the fourth decade of life, the curve demonstrates a relatively flat course until the eighth decade of life and a decline thereafter. The male-to-female ratio in individuals 80 years of age or older is 1.58:1 [c/f census 0.88:1].

**P-to-P**: Of the 150,086,257 (Table 1: C3) unique individuals tested, 8,741,043 (Table 1: C4) (5.82%) were tested more than once while P-to-P interval data was available in only 433,496 (0.29%) individuals. The mean P-to-P interval was 9.5 ± 9.9 days, while the maximum P-to-P was 238 days. The P-to-P duration was less than 1 week in nearly one-half (48%; *n* = 208,706) of the individuals while it was within 4 weeks in 97.26% (*n* = 421,600) of the individuals (Table 3).

**Table 3:** P-to-P interval data for COVID-19 from India (until December 29, 2020).

**P to P**	**Proportion**	**Mean (days)**	**Median (days)**	**SD (days)**
Any P-to-P duration	100.00% (*n =* 433,496)	9.5 days	7.0 days	9.9
P-to-P ≤ 4 weeks	97.26% (*n =* 421,600)	8.3 days	7.0 days	5.6
1 week < P-to-P ≤ 4 weeks	49.1% (*n =* 212,894)	12.7 days	9.0 days	4.3
**Time**	**P to P (% of total)**	**# P to P**
Within 1 Week: <7 days	48.14%	208,706
Within 2 Weeks; <14 days	83.81% (+36%)	363,332
Within 3 Weeks	94.70% (+11%)	410,537
Within 4 Weeks	97.26% (+3%)	421,600
Within 5 Weeks	98.25% (+1%)	425,903
More than/ equal to 28 days	2.74%	11,896
More than/equal to 6 weeks	1.24%	5,355
More than/equal to 8 weeks	0.71%	3,064
**Total**		**433,496**

**Figure 5:**
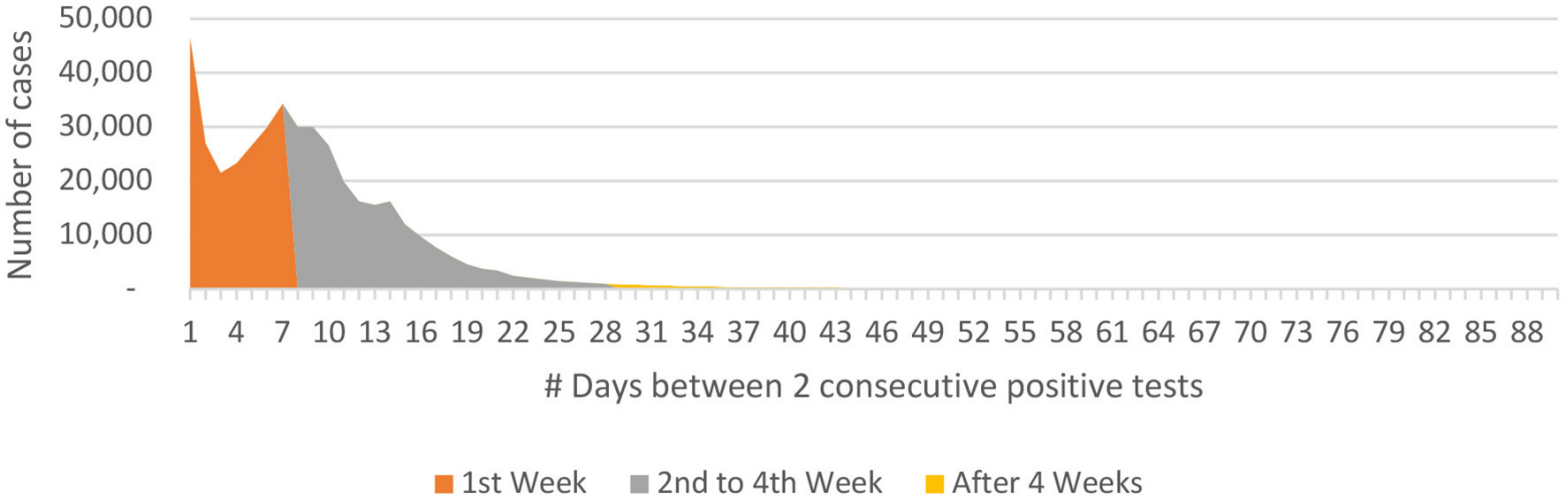
P-to-P interval data for COVID-19 from India (until December 29, 2020).

***The mean P-to-P duration was 12.7 days*** ***(+ 4.3 days) for*** ***individuals in whom the P-to-P duration was more than 1 week and up to 4 weeks***. There were only 1.24% (*n* = 5,355) and 0.71% (*n* = 3,064) of the individuals in whom the P-to-P interval was more than 6 and 8 weeks, respectively (Figure 5).

**Figure 6:**
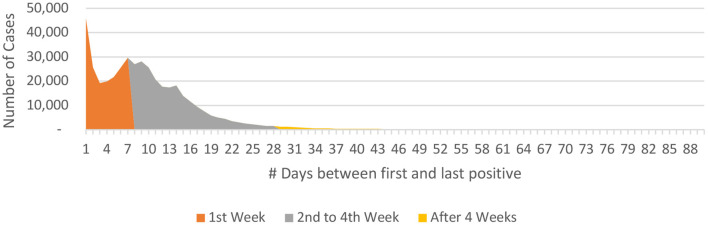
P-to-P_last_ interval data for COVID-19 from India (until December 29, 2020).

**P to P**_**last**_**:** The mean P-to-P_last_ interval was 10.4 + 10.5 days, the longest being 238 days. The P-to- P_last_ duration was less than 1 week in 43.53% (*n* = 188,718) individuals while it was within 4 weeks in 96.46% (*n* = 418,160) individuals (Table 4). ***The mean P-to-P***_***last***_ ***duration*** ***was 13.3 days (+4.6 days) for*** ***individuals in whom the P-to-P***_***last***_ ***duration was more than 1 week and up to 4 weeks*** (Figure 6). There were 3.54% (*n* = 15,336), 1.48% (*n* = 6,399) and 0.79% (*n* = 3,406) of the individuals in whom the P-to-P_last_ interval was more than 4, 6, and 8 weeks, respectively.

**Table 4:** P-to-P_last_ interval data for COVID-19 from India (until December 29, 2020).

**P to P_last_**	**Proportion**	**Mean (days)**	**Median (days)**	**SD (days)**
Overall	100%	10.4	7.0	10.5
P-to-P_last_ < 4 weeks	97.3% (*n =* 421,600)	9.0	7.0	6.0
1 week < P-to-P_last_ < 4 weeks	49.1% (*n =* 212,894)	13.3	9.0	4.6
**Time**	**P to P**_last_ **(% of total)**	**# P to P**_last_
Within 1 Week	43.53%	188,718
Within 2 Weeks	79.32% (+36%)	343,834
Within 3 Weeks	92.76% (+13%)	402,125
Within 4 Weeks	96.46% (+4%)	418,160
Within 5 Weeks	97.87% (+1%)	424,260
More than/equal to 28 days	3.54%	15,336
More than/equal to 6 weeks	1.48%	6,399
More than/equal to 8 weeks	0.79%	3,406
**Total**		**433,496**

**Figure 7:**
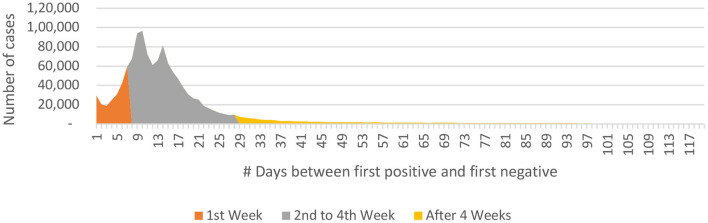
P-to-N interval data for COVID-19 from India (until December 29, 2020).

**P to N:** The mean difference between the first positive and the first negative was 18 ± 19.9 days, the maximum being 274 days. The P-to-N duration was less than 1 week in 18% (*n* = 225,708) of the individuals while it was within 4 weeks in 88% (*n* = 1,137,812) individuals (Table 5). ***The mean P-to-N duration was 14.2 days*** ***(+4.9 days) for individuals*** ***in whom the P-to-N***_***t***_ ***duration was more than 1 week and up to 4 weeks*** (Figure 7).

**Table 5:** P-to-N interval data for COVID-19 from India (until December 29, 2020).

**P to N**	**Proportion**	**Mean (days)**	**Median (days)**	**SD (days)**
Overall	100%	18.0	10.0	19.9
P-to-N < 4 weeks	88.44% (*n =* 1,137,812)	12.3	10.0	5.9
1 weeks < P-to-N < 4 weeks	70.90% (*n =* 912,104)	14.2	10.0	4.9
**Time**	**P to N (% of total)**	**# P to N**
Within 1 Week	17.54%	225,708
Within 2 Weeks	59.43% (+41%)	764,543
Within 3 Weeks	81.53% (+22%)	1,048,925
Within 4 Weeks	88.44% (+7%)	1,137,812
Within 5 Weeks	91.43% (+3%)	1,176,306
More than/equal to 28 days	11.56%	148,699
More than/equal to 6 weeks	6.97%	89,634
More than/equal to 8 weeks	5.00%	64,299
**Total**		**1,286,511**

**Figure 8:**
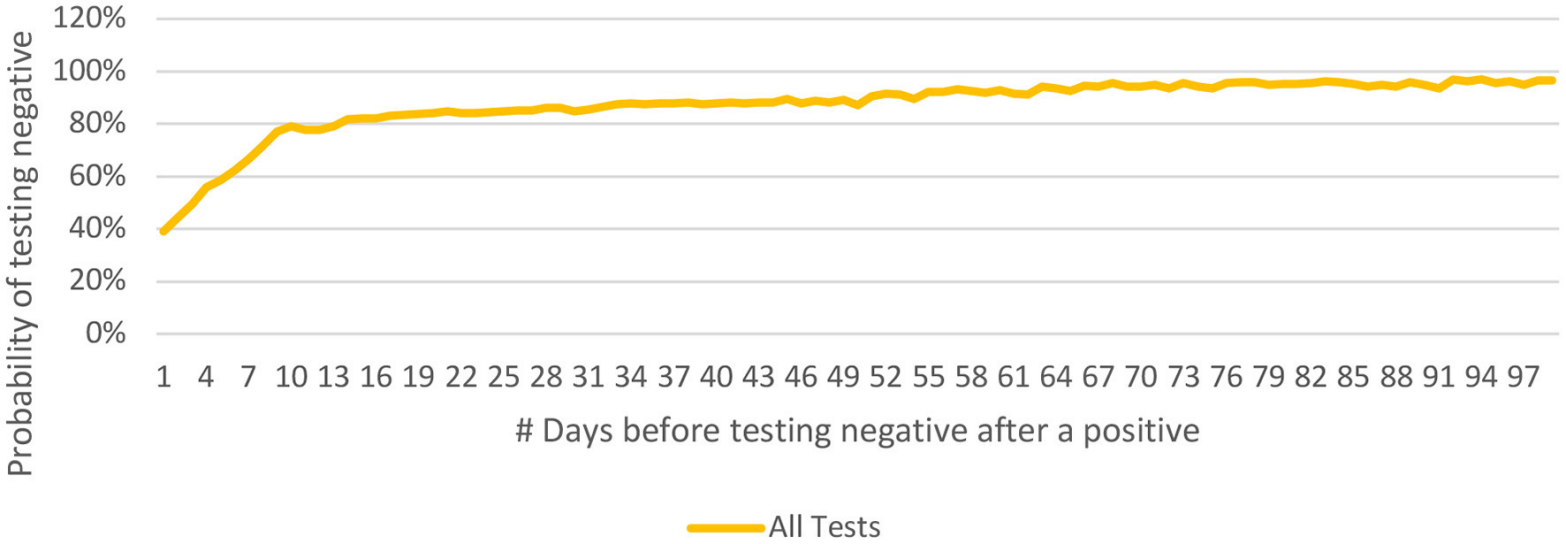
Probability of testing negative 'n' days after testing positive [P(n)] and its longitudinal spread for COVID-19 from India (until December 29, 2020).

The **Probability of Testing Negative 'n' Days After Testing Positive [P(n)]** was calculated as 67, 79, 82, 85, and 86% at 7, 10, 14, 21 and 28 days respectively after the first positive test (Figure 8).

**Figure 9:**
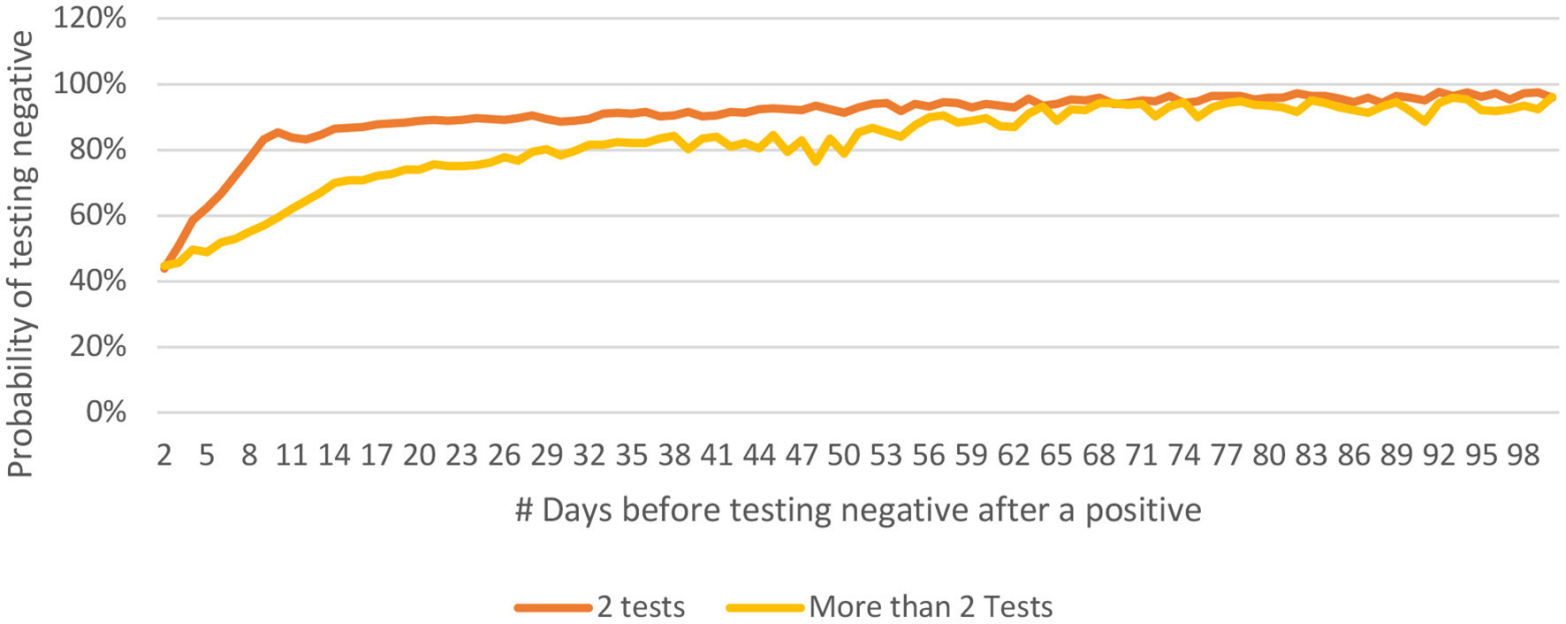
Distribution of P(n) for 2 tests only vs for cases with more than 2 tests for COVID-19 from India (until December 29, 2020).

**Figure 10:**
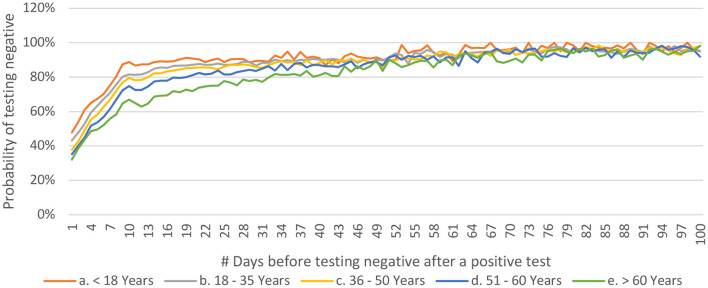
Distribution of P(n) across age-groups for COVID-19 from India (until December 29, 2020).

People who have been tested only twice are more likely to test negative earlier as compared to those who have undergone more than two tests (Figure 9). This may be related to the general understanding that a person who continues to be symptomatic is likely to get himself/ herself tested repeatedly. It was observed that the younger people tested negative earlier than their older counterparts (Figure 10).

## Discussion

To the best of our knowledge, this is the largest report on the coronavirus pandemic to date from India based on data accrued prospectively from 170,914,170 tests (Table 1: A0) conducted over a period of one year. The overall positivity rate for the disease was 6.60%. It was observed that the positivity rate was 4 times higher among those individuals who were tested more than once (17). This reflects human behavior as much as the need generated by the prevailing treatment protocols or lack of knowledge. Those individuals who test positive are likely to repeat their tests for a second confirmation, occupational, or social pursuit for a negative report and it is also possible that the exposure risk of these individuals is higher and may be the reason for more frequent testing. A group of patients seeking treatment for unrelated conditions such as acute cholecystitis was reported positive while a COVID-19 test was conducted as per protocol. A negative report was awaited prior to elective-treatment for their primary condition.

A significant majority of the patients (87.31%) have never been reported negative after an initial positive report. While the pandemic was unfolding itself, the understanding of the disease and the guidelines for management were evolving too. Sparing the initial months of the pandemic, a negative test has never been considered a criterion to consider a patient cured.

Individuals belonging to older groups accounted for a higher proportion of population tested. The working age-group (herein, 18–60 years) which comprises nearly one-half of the population constituted 80% of the caseload. An age-based analysis has highlighted the ***vulnerability of the elderly (more so, with pre-existing comorbidity)***: individual >60 years of age (~7% of total- population) accounted for 14% of the caseload compared to those < 18 years of age (~37% of total- population) accounting for 7.5% of the caseload. This has also been supported through existing studies (7, 18–22). The positivity rates were lowest in infants and children, possibly due to a better immune response, lower incidence of comorbidities, indoor habitat, and limited socialization (23, 24).

Schools were closed and so were the parks or other amusement avenues. The bread-earner, however, was vulnerable to infection.

The pandemic management in India was peculiar with an early declaration of complete lockdown (March 24, 2020) (25, 26). Unlock was initiated in June 2020 and was gradual and phasic (25). While the younger age-groups (< 18 years) were relatively stable on the timeline, the age-group >50 years witnessed a steep rise in proportion beyond September 2020 which was compensated by a corresponding decline for the 18–50 years age-group. The increase in proportion of cases for >50 years can be explained by the fact that lockdowns were relaxed which led to greater exposure to the virus of an earlier unexposed group. While at the same time since schools and colleges remained closed, the exposure of age-group < 18 years remained relatively similar.

The bimodal distribution of the positivity rate (Figure 3A) could be related to the disease, immunity differences across different age-groups (7, 18–21) with the children having a better immune response against the virus, higher likelihood of a coexisting morbidity in the elderly age-groups, increased socialization in the older groups, and possible ignorance and lack of awareness to preventive measures such as social distancing and hand-hygiene. Similar to the observation in Figure 3A, we see in Figure 3B that this difference in positivity over time remains consistent with people above the age of 50 years having a higher positivity rate as compared to their younger counterparts.

Congruent to our understanding of the local customs and societal norms, males have been tested more than females (1.5:1) and are nearly twice as likely (1.94:1) to undergo multiple tests. Preferential outdoor-jobs and outdoor-psychology grants them easy access to testing centers and higher vulnerability. The ratio of positives was higher in men too (1.73:1) and was higher than the testing ratio (1.5:1) implying a higher positivity. This has been implicated to gender-specific behavior with a higher involvement with smoking and drinking, irresponsible behavior toward preventive measures such as use of facemasks and frequent hand-hygiene, biological differences with a higher expression of angiotensin converting enzyme-2 receptors, immunological differences mediated by the sex hormones and the X chromosome, vulnerability to exposure due to office, outdoor activities and socialization, and easy access to testing centers (19, 27, 28).

The gender differences in statistics were not apparent in the younger age-groups: the male-to-female case ratio was nearly 1:1 in neonates as compared to 1.7:1 across the entire population; the outlined male-specific factors were inoperative in this age-group. The proportion of affected females has risen with easing lockdown-restrictions. The male-to-female ratio of 1.98:1 (April-May 2020) depicted a progressive trend favoring females with a plateau at 1.6:1 (October-December 2020). Another peculiar aspect of the male-to-female ratio was reflected by the rising in proportion of affected males with age (29). The male-to-female ratio was 1.18:1 in children < 10 years of age and showed a progressive trend toward 2:1 in 30–39 years of age. This is reflective of the increased outdoor and social behavior of males compared to females in the context of Indian society. Other factors as outlined ahead such as responsible behavior and differences pertaining to lifestyle, immunity, genetic, and hormonal composition may also become active or more pronounced with age. The curve plateaus for the next four decades of life. The reverse trend, thereafter, suggests amelioration of gender-based differences beyond a certain age and a longer life-expectancy for females (27, 30).

**P-to-P interval** was studied to estimate a) the duration for which a person may test positive after infection, b) the frequency with which the test has been repeated in those diagnosed positive, and c) calculate the probability of a repeat positive test after 'n' days of a positive test. The P-to-P interval assumes maximum relevance when it is more than 1 week and less than 4 weeks: 12.7 days±4.3 days (31, 32). *The testing sequence is less likely to represent the true positivity after infection when P-to-P*>*4 weeks*. A minority of patients continued to test positive for 6 and 8 weeks after the first positive: 1.2 and 0.7% respectively.

**P-to-P**_**last**_ interval was studied to a) estimate the maximum duration for which a person may continue to test positive after initial infection, and b) identify the complex cases. P-to-P_last_ may be considered as a proxy for viral shedding; the data reflects that viral shedding may persist for at least 4 and 6 weeks in 3.5 and 1.5% patients, respectively. However, persistence of viral- shedding may not be interpreted as active-infection; the reports indicate the anatomic presence of the viral components in the specimens. Incidence of a positive test was reduced to 0.8% after 8 weeks.

The **P-to-N interval** was analyzed to decipher a) the time to cease viral shedding, b) the probability of testing negative 'n' days after the first positive test. The actual cessation of viral shedding should happen somewhere between the P-to-P_last_ and the P-to-N intervals. ***Considering that the average*** ***P-to-P***_***last***_ ***was 13.3 days, and the average*** ***P-to-N*** ***was 14.2 days, it may be safe to advise that a person*** ***seeking a negative report should repeat the test at least 14 days after the first positive*** ***instance***. This current data may have global implications for formulation of policies in relation to future management of the current pandemic and the statistical model used herein may be used to analyze the data and generate logical conclusions for a future pandemic.

The probability distribution curve (for testing negative 'n' days after the first positive instance) rises steeply for 10 days when 79% of the patients tested negative and flattens thereafter. The quarantine restrictions for a positive patient may therefore be eased at 10 days after the first- positive test, provided the patient is asymptomatic. Caution may be exercised while interpreting these observations; the data is limited by the inadvertent delay between the disease-onset and first positive test. Similarly, a positive test beyond 4 weeks (14% of cases) may not necessarily indicate active infection or infectivity.

Furthermore, the observations that younger patients test negative earlier (Figure 10) with no such discrimination across the gender-stratification may be significant for the policymakers and epidemiologists (18, 33, 34). It should be noted that 80% of the population < 18 years starts testing negative in less than 10 days of the first positive test, whereas 80% of the population over the age of 60 years starts testing negative only after 30 days of the first positive test. This might highlight the fact that younger population is able to mount a better response against the virus and may also help in deciding the time to retest a patient in hopes of getting a negative test. One of the papers from Madrid estimated the days to negative PCR as 14 ranging between 12 to 17 in healthcare workers (35). Another paper from Pakistan, estimated the range to be between 8.54 to 13.64 depending on the severity of the disease (36).

The P-to-P, P-to-P_last_ and P-to-N intervals must however be interpreted with caution; the first positive is not the same as the onset of infection or infectivity. Similarly, the second positive test or the negative test does not hallmark the end of infection or disease positivity at that point of time. Also, a patient who tested negative, say 5 weeks later, may have stopped shedding the virus earlier. The data P-to-P_last_ and P-to-N must be interpreted in synchronicity; a large P-to-P_last_ is significant and so is a small P-to-N while the vice-versa may be equally insignificant. The number °f days between P_last_ and N (N_first_ in case of more than one negative) has prognostic implications and needs further evaluation.

## Data availability statement

The datasets presented in this article are not readily available because the funding agency has restricted the access to this data. Requests to access the datasets should be directed to secy-dg@icmr.gov.in.

## Ethics statement

This study has been approved by Director General, Indian Council of Medical Research. The study does not involve taking individual patient consent as no patient was exclusively sampled for the study and anonymized data was used for the analysis.

## Author contributions

HS has conceptualized the research. JK and RL have extracted and analyzed the data. PG and RL are lead authors of the manuscript. SK and MB have provided their valuable inputs in the analyzing the results. All authors have read and approved the manuscript.

## Funding

The study has been funded by Indian Council of Medical Research, New Delhi (No. ISRM/14(01)/TF/Genome/2017).

## Conflict of interest

The authors declare that the research was conducted in the absence of any commercial or financial relationships that could be construed as a potential conflict of interest.

## Publisher's note

All claims expressed in this article are solely those of the authors and do not necessarily represent those of their affiliated organizations, or those of the publisher, the editors and the reviewers. Any product that may be evaluated in this article, or claim that may be made by its manufacturer, is not guaranteed or endorsed by the publisher.
